# Antimicrobial Potency of Fmoc-Phe-Phe Dipeptide Hydrogels with Encapsulated Porphyrin Chromophores Is a Promising Alternative in Antimicrobial Resistance

**DOI:** 10.3390/biom14020226

**Published:** 2024-02-16

**Authors:** Chrysanthi Pinelopi Apostolidou, Chrysoula Kokotidou, Varvara Platania, Vasilis Nikolaou, Georgios Landrou, Emmanouil Nikoloudakis, Georgios Charalambidis, Maria Chatzinikolaidou, Athanassios G. Coutsolelos, Anna Mitraki

**Affiliations:** 1 Department of Materials Science and Technology, University of Crete, Voutes Campus, 71003 Heraklion, Greece; chrysana@materials.uoc.gr (C.P.A.); chkokoti@hotmail.com (C.K.); plataniavarvara@yahoo.com (V.P.); 2 Institute of Electronic Structure and Laser (IESL) FORTH, 70013 Heraklion, Greece; 3 Laboratory of Bioinorganic Chemistry, Department of Chemistry, University of Crete, Voutes Campus, 70013 Heraklion, Greecegiorgosland@hotmail.com (G.L.); nik21man@gmail.com (E.N.); gcharal@uoc.gr (G.C.); 4 Theoretical and Physical Chemistry Institute, National Hellenic Research Foundation, 48 Vassileos Constantinou Ave., 11635 Athens, Greece

**Keywords:** porphyrins, hydrogels, self-assembling peptides, antimicrobial activity, encapsulation, controlled release, photodynamic inactivation

## Abstract

Antimicrobial resistance (AMR) poses a significant global health risk as a consequence of misuse of antibiotics. Owing to the increasing antimicrobial resistance, it became imperative to develop novel molecules and materials with antimicrobial properties. Porphyrins and metalloporphyrins are compounds which present antimicrobial properties especially after irradiation. As a consequence, porphyrinoids have recently been utilized as antimicrobial agents in antimicrobial photodynamic inactivation in bacteria and other microorganisms. Herein, we report the encapsulation of porphyrins into peptide hydrogels which serve as delivery vehicles. We selected the self-assembling Fmoc-Phe-Phe dipeptide, a potent gelator, as a scaffold due to its previously reported biocompatibility and three different water-soluble porphyrins as photosensitizers. We evaluated the structural, mechanical and *in vitro* degradation properties of these hydrogels, their interaction with NIH3T3 mouse skin fibroblasts, and we assessed their antimicrobial efficacy against Gram-positive *Staphylococcus aureus* (*S. aureus*) and Gram-negative *Escherichia coli* (*E. coli*) bacteria. We found out that the hydrogels are cytocompatible and display antimicrobial efficiency against both strains with the zinc porphyrins being more efficient. Therefore, these hydrogels present a promising alternative for combating bacterial infections in the face of growing AMR concerns.

## 1. Introduction

Antimicrobial therapy was supported by the discovery of antibiotics in the 1940s and is one of the greatest achievements of modern medicine. However, there has been overuse and misuse of antibiotics over the years, resulting in antimicrobial resistance (AMR) [1]. Due to the increasing antimicrobial resistance, it became imperative for scientists to develop novel molecules and materials with antimicrobial properties [2]. Such materials include natural [3] and designed antimicrobial peptides [4,5], metal nanoparticles [6], and porphyrins [7,8]. Porphyrins and metalloporphyrins represent a class of compounds that play a critical role against pathogens because they are low-toxicity molecules and can effectively kill the pathogens through photodynamic activation [9,10,11,12]. Upon light activation, porphyrinoids absorb photons and form a long-lived triplet excited state via a short-lived singlet excited state. From the triplet excited state, the porphyrinoids can form reactive oxygen species (ROS), following two different pathways. First, through an electron-transfer process with a biomolecule, it can form radicals and radical ions, which, after interaction with oxygen, can generate oxygenated products such as superoxide ions (type I reaction). On the other hand, it can participate in an energy transfer process, resulting in the formation of reactive singlet oxygen (type II reaction).

The reactive oxygen species can cause damage to cellular components, including proteins, lipids, and nucleic acids. In the case of bacterial cells, this oxidative stress can lead to the lysis of the cell wall. The cell wall is crucial for maintaining the structural integrity of bacterial cells, and its disruption can ultimately result in cell death [13].

Therefore, porphyrins constitute promising photosensitizers (PSs) for use in biomedical applications [14]. Further modifications, producing, for example, cationic porphyrin derivatives, may result in higher toxicity against bacteria [15]. Metalloporphyrins, especially the zinc-containing ones, have been extensively studied in the last decade due to their photophysical and photochemical properties, with promising results in antimicrobial photodynamic inactivation (aPDI) in bacteria and other microorganisms, including fungi and viruses [8]. Moreover, as Zn-porphyrins display low dark toxicity to mammalian cells in culture, suitably designed metalloporphyrins may be a promising alternative to antibiotics in cases of antibiotic resistance [16].

**Scheme 1:**
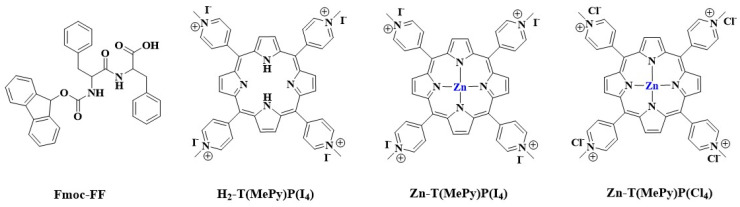
Chemical structures of the peptide Fmoc_-FF and the three porphyrin-based molecules synthesized in this study: H_2_-T(MePy)P(l_4_); Zn-T(MePy)P(I_4_); and Zn-T(MePy)P(Cl_4_).

Formulations of the abovementioned antimicrobial agents, e.g., ointments, creams, etc., are important especially when destined for topical use, for example, in wound healing. Hydrogels have been proposed as an alternative wound-healing formulation in recent years. Hydrogels are polymeric networks that can maintain their structure and absorb a large amount of water [17]. The main advantage of hydrogels is that they provide a moist and beneficial environment at the wound site, which promotes tissue regeneration by granulation and re-epithelialization [18]. Hydrogels composed of chitosan, hyaluronic acid, and other carbohydrate-based hydrogels have been extensively studied as materials targeted for wound-healing applications [19]. Self-assembling peptides are another class of biomolecules that find biomedical applications due to their ability to self-organize into supramolecular structures, in the form of, for example, fibers, nanotubes, or hydrogels. These supramolecular structures are biocompatible and form under mild conditions, thanks to weak but significant secondary and non-covalent interactions [20]. The study of these structures finds great appeal in the fields of materials science, nanotechnology, and bio-medicine [21,22,23,24,25,26,27,28]. As they are ultrashort, i.e., less than six amino acid peptides, they form minimalistic building blocks that self-assemble into a variety of nanostructures, including hydrogels [29,30,31]. These ultrashort self-assembling peptide hydrogels are biocompatible and biodegradable and have recently gained interest for applications for tissue regeneration and wound healing [32,33,34]. The Phe-Phe (FF) dipeptide, when protected at the N-terminus with the Fmoc (fluorenyl-methoxycarbonyl) group, represents a minimal building block that can self-assemble and form hydrogels at the physiological pH [35]. Numerous studies have demonstrated the capability of the Fmoc-FF dipeptide, shown in Scheme 1, to self-assemble into hydrogels that can act as tissue engineering scaffolds or as reservoirs for the encapsulation and controlled release of model small molecules or drugs [36,37]. Therefore, Fmoc-FF hydrogels, in combination with their anti-infective properties, were deemed suitable scaffolds for biomedical applications such as controlled drug delivery [38]. We have recently encapsulated the water-soluble porphyrin H_2-_T(MePy)P(I_4_), shown in Scheme 1, into Fmoc-FF hydrogels; the delivery of the porphyrin through the self-assembling hydrogel accelerated the healing of experimental wound defects *in vivo* [39].

One important issue in the wound-healing process is bacterial infections, either by external pathogens or bacteria from the skin microbiota [40]. The present study focuses on evaluating the antibacterial potential of Fmoc-FF dipeptide hydrogels encapsulating porphyrin chromophores, in an effort to develop a bifunctional gel combining antimicrobial properties with their recently demonstrated wound-healing properties. Thus, we performed the encapsulation of porphyrin derivatives into the hydrogel-forming Fmoc-FF scaffold. In detail, we focused on one metal-free cationic porphyrin, and its corresponding two zinc metalloporphyrin complexes, shown in Scheme 1. The choice of porphyrins stemmed from their distinctive characteristics, particularly their solubility in aqueous solutions, photostability, robust absorption capacity, and inherent ability to produce reactive oxygen species [14,41]. On the other hand, the Fmoc-FF is well-established for forming highly stable hydrogels [42]. Initially, we focused on the structural characterization of the peptide–porphyrin-containing hydrogels using field emission scanning electron microscopy (FESEM). Recognizing that the encapsulated porphyrins might alter the Fmoc-FF hydrogel’s mechanical and stability properties, we tested the mechanical and *in vitro* degradation properties of the porphyrin-containing hydrogels. Moreover, a hydrogel should be non-toxic to mammalian cells to be deemed suitable for therapeutic applications. The cytotoxicity of the Fmoc-FF hydrogel without any loaded porphyrin and of the Fmoc-FF loaded with porphyrins was assessed by means of the MTT assay in NIH3T3 mouse skin fibroblasts. Finally, to further evaluate the eventual therapeutic potential of these hydrogels, we evaluated their antimicrobial efficacy against Gram-positive *Staphylococcus aureus* (*S. aureus*) and Gram-negative *Escherichia coli* (*E. coli*) bacteria.

## 2. Materials and Methods

### 2.1. Materials

Fmoc-Phe-Phe peptide powder with a purity greater than 95% was purchased from Bachem (Bubendorf, Switzerland). The porphyrins H_2-_T(MePy)P(I_4_), Zn-T(MePy)P(I_4_), and Zn-T(MePy)P(Cl_4_) were synthesized as previously described [43]. The NIH3T3 cell line was cultured at 37 °C, 5% CO_2_ in DMEM (Gibco, Thermo Fisher Scientific, Waltham, MA, USA) medium supplemented with 10% fetal bovine serum (Gibco, Thermo Fisher Scientific, Waltham, MA, USA) and 50 μg/mL gentamycin (Applichem, Darmstadt, Germany). Thiazolyl blue tetrazolium bromide (MTT), isopropanol, dimethylsulfoxide (DMSO), ethanol, hexamethyldisilane (HMDS), and phosphate-buffered saline (PBS) were purchased from Sigma-Aldrich (St. Louis, MO, USA). Live/Dead cytotoxicity assay for mammalian cells was purchased from Thermo Fisher Scientific (Waltham, MA, USA). Tissue culture (TC) inserts with a 0.4 μm pore size were purchased from Sarstedt (Nümbrecht, Germany).

### 2.2. Methods

#### 2.2.1. Fabrication of Peptide Hydrogels

The Fmoc-FF hydrogels with the encapsulated porphyrins were fabricated following the solvent-switch method [37]. The peptide powder was first dissolved into a “good solvent”—(ethanol); subsequently, a “bad solvent”—(water) was added to trigger the self-assembly process and hydrogel formation. The Fmoc-FF peptide hydrogel was therefore prepared in a solution of ethanol/H_2_O with a ratio 25:75 *v*/*v* at a peptide concentration of 5 mg/mL. More specifically, the peptide powders were weighed and dissolved in 250 μL of ethanol with concomitant heating at 60 °C in a water bath and periodical sonication. A total of 750 μL of sterilized water was added into the dissolved peptide solution to initiate the self-assembly process. The mixtures were kept at room temperature for 1 h to allow sufficient time for the completion of self-assembly. For the fabrication of the hydrogels encapsulating the metalloporphyrins, the same method was applied for dissolving the peptide powder in 250 μL of ethanol. Metalloporphyrins were dissolved in 750 μL of sterile distilled water at a final concentration of 0.3 mM. The molar ratio of peptide-to-porphyrin was 30 to 1. The metalloporphyrin solution was mixed with the dissolved peptide solution with smooth pipetting. The self-assembly into fibrils was triggered instantaneously. The mixture was placed at room temperature for 1 h to allow the completion of the self-assembly process.

#### 2.2.2. Field Emission Scanning Electron Microscopy (FESEM)

The hydrogel morphology was observed by using field emission-SEM (FESEM). Samples for FESEM analysis were prepared by depositing 10 μL from each self-assembled hydrogel onto a 12 mm cover glass. The samples were left to air-dry overnight. Dried samples were sputter-coated with a 10 nm thick layer of Au (Baltec SCD 050) and observed directly. FESEM experiments were performed using a JEOL JSM 7000F operating at 15 kV.

#### 2.2.3. Mechanical Characterization of the Scaffolds

The mechanical properties of the various Fmoc-based hydrogels were evaluated by uniaxial compression tests as previously described [44]. Hydrogel samples with a diameter of 1.2 cm and a height of 2 cm were employed. The compressive stress–strain measurements were performed in at least triplicates by means of a mechanical testing system equipped with a 50 N loading cell (CellScale, Waterloo, ON, Canada) in the air at room temperature. Compression was applied at a deformation speed of 15 mm/s up to 60% strain. The Young’s modulus of the samples was determined as the slope in the linear elastic deformation region, corresponding to 5-20% strain of the stress–strain diagrams.

#### 2.2.4. MTT Cell Proliferation Assay

NIH3T3 cells with concentrations of 6 × 10^4^ cells/well were cultured in a 24-well plate for 24 h. A total of 100 μL of Fmoc-FF peptide hydrogel was added into a hanging basket with a filter size of 0.4 μm and it was attached onto the well. This indirect—no contact—cytotoxicity test method aimed to evaluate whether the Fmoc-FF hydrogel, while in solution, releases potential cytotoxic residuals that may harm the fibroblast cell proliferation. Then, 1 mL of DMEM medium was added into the well to ensure sufficient contact with the bottom of the hanging basket. Cells that were not treated with the hydrogels served as the control. The well plate with the inserts was incubated for 48 h at 37 °C. Cell proliferation was measured with the MTT assay. After 48 h of incubation, the medium was replaced with 300 μL of fresh medium and 30 μL of MTT (5 mg/mL) dissolved in PBS. The cells were incubated for 4 h to allow the development of the purple formazan products and the MTT/culture medium was substituted with 300 μL of an isopropanol/DMSO 1:1 solution. The formazan crystals were allowed to dissolve for 15 min at 37 °C. The absorbance was measured at 570 nm in a Synergy HTX BioTEK Plate Reader. The cytotoxicity of the peptide hydrogels encapsulating metalloporphyrins, were not examined with this method, since the porphyrins have an inherent absorbance at this wavelength that may interfere with the MTT measuring absorbance.

#### 2.2.5. Fixation and Dehydration of Cells Attached on Peptide Hydrogel Scaffolds

A direct-contact method was employed to examine the potential cytotoxicity of the peptide hydrogel when used as a scaffold for cell proliferation. This method was used to examine whether fibroblasts can attach and proliferate on top of the peptide hydrogel area. Peptide hydrogels without and with encapsulated metalloporphyrins were added at a quantity of 50 μL on top of a 10 mm circular cover glass which was placed inside a 24-well plate. The hydrogel material was spread to cover the whole area of the glass. Next, 6 × 10^4^ of NIH3T3 cells were added onto the hydrogel. The plates were incubated at 37 °C for 48 h. After 48 h, the medium was removed, and the attached cells were washed twice with 300 μL of PBS. Cell fixation was performed by adding 300 μL of 4% paraformaldehyde to each well for 15 min. Paraformaldehyde was removed and the cells were washed again twice with 300 μL of PBS. The cells were further incubated for 5 min each time with increasing concentrations of ethanol (30, 50, 70, 90, and 100% dry ethanol) to dehydrate the samples. Finally, 200 μL of HMDS was added to each well and left to evaporate overnight. The cover glass was removed with a tweezer and placed on top of a carbon tape for further processing and observation with FESEM.

#### 2.2.6. Live/Dead Cytotoxicity Assay

Another direct-contact cytotoxicity test method was used to evaluate cell proliferation when the peptide hydrogel was added into the same medium where cells incubate. This method aims to give information on the potential application of the peptide hydrogels as injectables in the skin or plasma. A total of 50 μL of the peptide hydrogels was added at the bottom of each well of a 24-well plate. Then, 1 × 10^5^ NIH3T3 cells were added on top of the solidified hydrogel and the plate was incubated for 24 h at 37 °C. The staining solution was prepared by adding 5 µL calcein AM and 20 µL ethidium homodimer-1 to 10 mL of PBS. After the incubation period, the medium was removed and 200 μL of the staining solution was added directly to cells. For efficient staining, the cells were left at RT for 30 min. The stained live and dead cells were observed with a fluorescent microscope by using a 494/517 nm filter for calcein AM for live cells and a 528/617 nm filter for ethidium homodimer-1 for the dead cells.

#### 2.2.7. *In Vitro* Degradation of Hydrogels

The hydrogels comprised of Fmoc-FF and Fmoc-FF with encapsulated porphyrins were formed as described above. The weight loss of the prepared hydrogels was used to measure the degradation rate of hydrogels. The prepared hydrogels (300 μL) were immersed in 2 mL of PBS solution (pH = 7.4) and incubated at 37 °C. At specific time points (1, 2, 7, 12, and 17 days), the hydrogels were removed from PBS, the remaining water was wiped off, and the mass of the remaining hydrogel was measured. The mass loss of the hydrogels (*n* = 3) was calculated using Equation (1):

(1) $$\mathrm{Mass}\;\mathrm{loss}\;\left(\%\right)\;=\frac{\mathrm{mi}-\mathrm{mt}}{\mathrm{mi}}\times 100$$

where mi is the initial mass of hydrogels and mt is the residual mass after removal from the solution.

#### 2.2.8. Release Evaluation

The hydrogels were prepared as described above. A total of 500 μL of the prepared gels was incubated into cuvettes for 24 h at room temperature. Subsequently, 1 mL of water was placed on top of the gel at 37 °C and the water was replaced every 6 h hours. The amount of porphyrin released from the hydrogels in the water was quantified using UV–Vis spectrophotometry (UV-1700 PharmaSpec; Shimazdu Corporation, Kyoto, Japan) at a wavelength of 519 nm for the H_2_-T(MePy)P(I_4_) porphyrin and of 564 nm for the two metalloporphyrins, Zn-T(MePy)P(I_4_) and Zn-T(MePy)P(Cl_4_). All porphyrin release data were averaged over three measurements. The cumulative porphyrin release was calculated using Equation (2):

(2) $$Cumulative\;release\;\left(\%\right)\;=\frac{amount\;of\;drug\;released\;at\;time\;t+amount\;of\;drug\;released\;at\;time\;t-1}{initial\;amount\;of\;drug\;encapsulated\;in\;gel}\times 100$$

#### 2.2.9. Evaluation of Antimicrobial Activity

The samples were prepared as described above into 1 mL syringes. One colony of *E. coli* and one colony of *S. aureus* were left to grow in 5 mL of Luria Broth (LB) at 37 °C for 16 h. Then, 500 μL of the pre-culture was transferred into 50 mL of Luria Broth and incubated in a shaker (200 rpm) at 37 °C until the mid-log phase was reached (OD_600_ = 0.1). Then, the *E. coli* and *S. aureus* cells were diluted 6 times and 600 μL was transferred into 2 mL Eppendorf tubes. Next, 100 μL of the prepared gels of Fmoc-FF, Fmoc-FF with H_2_-T(MePy)P(I_4_), Fmoc-FF with Zn-T(MePy)P(I_4_), and Fmoc-FF with Zn-T(MePy)P(Cl_4_), were transferred into the tubes with the *E. coli* and *S. aureus.* The samples were incubated for 5 h at 37 °C and subsequently irradiated under a visible light lamp (LED 10 mW·cm^−2^) for one hour accepting a light dose of 36 J·cm^−2^ under agitation at 100 rpm. Control samples were prepared and incubated for 6 h at 37 °C without light irradiation. The bacterial strain in the LB served as the negative control. After 6 h, the optical density at 600 nm was measured. As a reference sample, the LB broth without cells was used. Next, 100 μL was withdrawn from the negative control and from the encapsulated gel samples and was plated on Luria-agar plates after serial dilutions. The plates were incubated at 37 °C for 12–16 h, and bacterial colonies were counted to calculate colony forming units per milliliter (CFU/mL).

#### 2.2.10. Statistical Analysis

Statistical analysis was performed using ANOVA t-test in the GraphPad Prism version 8.3 software to evaluate significance of the differences among various hydrogels and the control. A *p*-value (*) < 0.05 was considered significant, ** *p* < 0.01, *** *p* < 0.001, **** *p* < 0.0001, ns denotes a statistically non-significant difference. Results are presented as mean ± SD.

## 3. Results

### 3.1. Structural and Mechanical Characterization of Porphyrin-Containing Fmoc-FF Hydrogels

#### 3.1.1. Structural Characterization of Hydrogels

**Figure 1:**
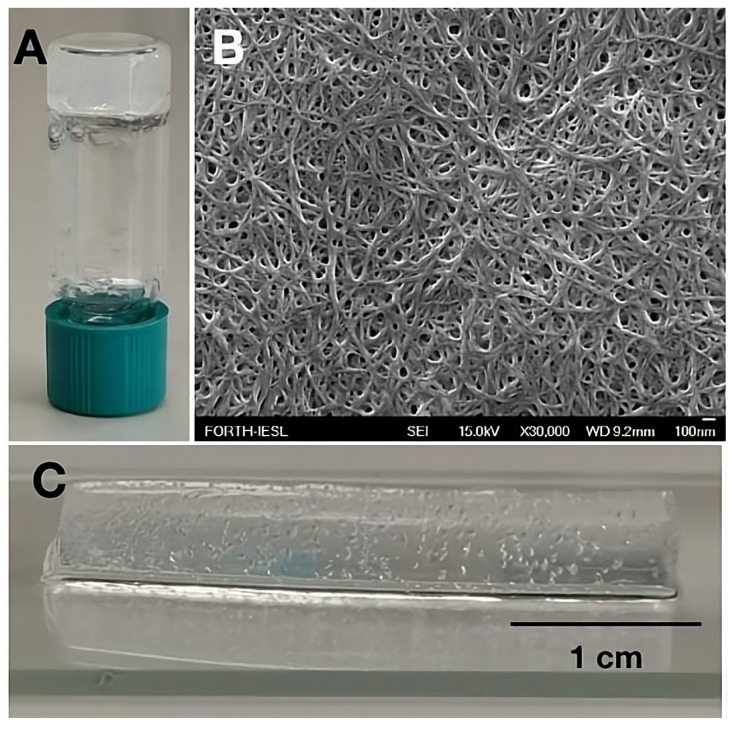
Fmoc-FF hydrogel. (**A**) The hydrogel in a glass vial placed upside down; (**B**) fibrillar formation as observed by FESEM (scale bar: 100 nm); (**C**) Fmoc-FF hydrogel shape integrity after being extruded from a syringe.

**Figure 2:**
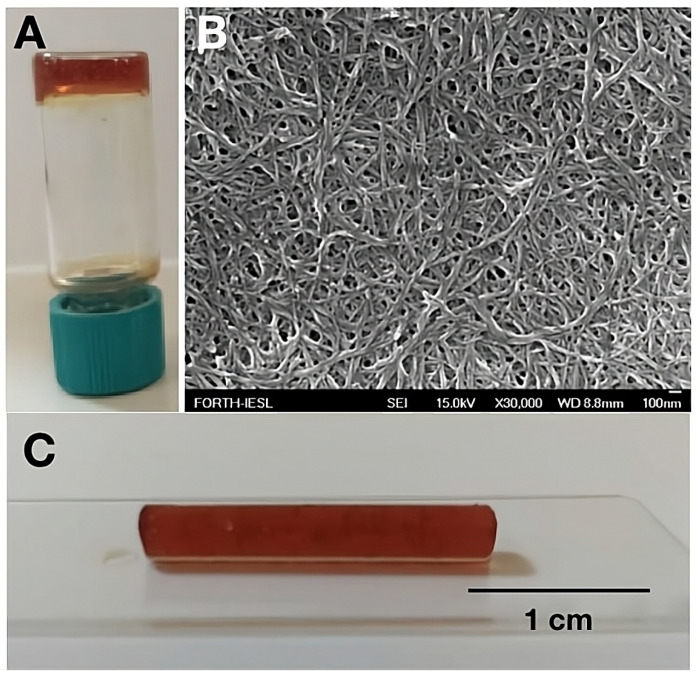
Fmoc-FF hydrogel encapsulating the H_2_-T(MePy)P(I_4_) porphyrin. (**A**) The hydrogel in a glass vial placed upside down; (**B**) fibrillar formation as observed by FESEM (scale bar: 100 nm); (**C**) Fmoc-FF-[H_2_-T(MePy)P(I_4_)], hydrogel shape integrity after being extruded from a syringe.

**Figure 3:**
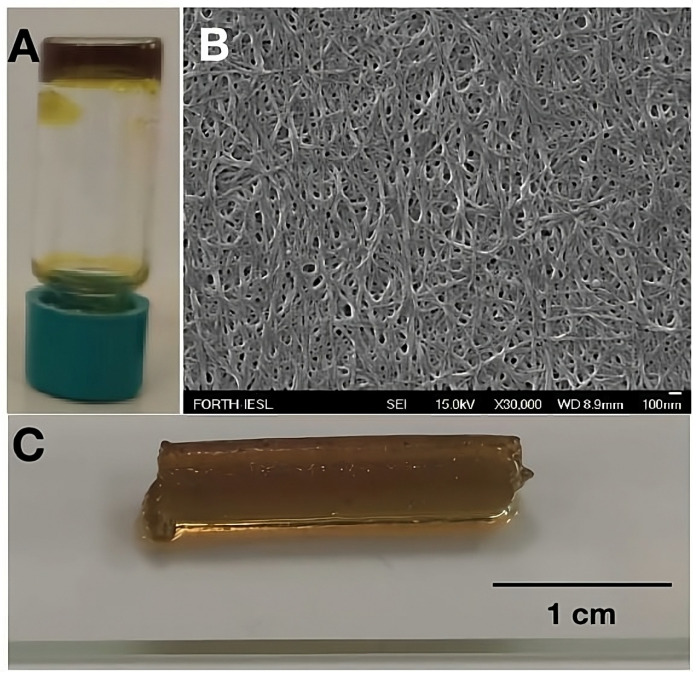
Fmoc-FF hydrogel encapsulating the Zn-T(MePy)P(Cl_4_) metalloporphyrin. (**A**) The hydrogel in a glass vial placed upside down; (**B**) fibrillar formation as observed by FESEM (scale bar: 100 nm); (**C**) Fmoc-FF-[Zn-T(MePy)P(Cl_4_)] hydrogel shape integrity after being extruded from a syringe.

**Figure 4:**
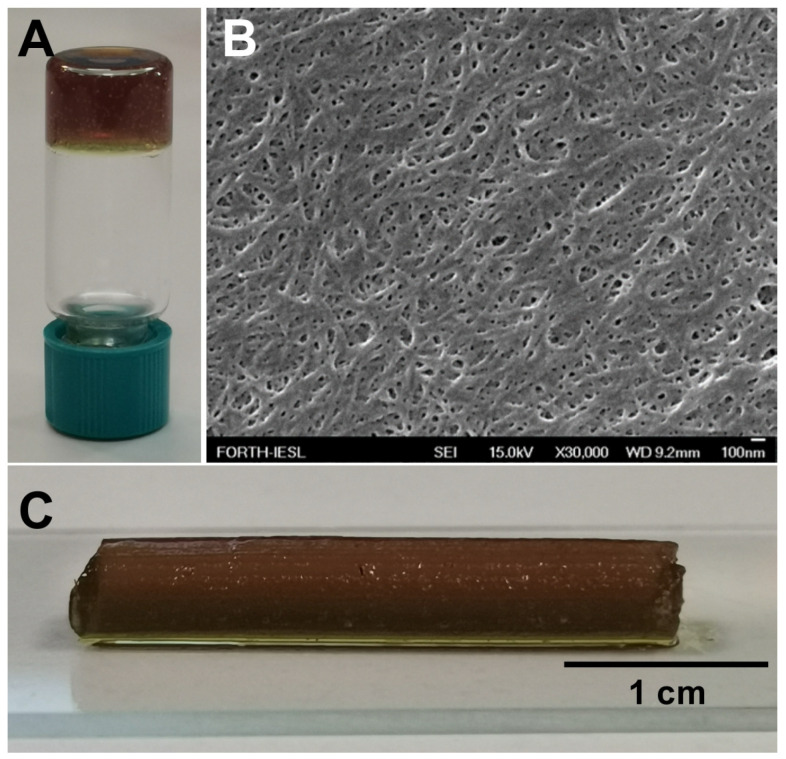
Fmoc-FF hydrogel encapsulating the Zn-T(MePy)P(I_4_) metalloporphyrin. (**A**) The hydrogel in a glass vial placed upside down; (**B**) fibrillar formation as observed by FESEM (scale-bar: 100 nm); (**C**) Fmoc-FF-[Zn-T(MePy)P(I_4_)] hydrogel shape integrity after being extruded from a syringe.

The Fmoc-FF peptides mixed with metalloporphyrins rapidly self-assemble into hydrogels of fibrillar morphology. The peptide powder, after being dissolved in ethanol solution and heat-treated, is mixed with an aqueous solution of porphyrins. The gel formation happens within a few seconds, and it is observed by the transition from a clear to a hazy viscous solution that subsequently becomes a clear self-supporting gel, indicating the completion of the self-assembly. When the hydrogel formation is complete, the vials containing the metalloporphyrin-loaded hydrogels are turned upside down to evaluate the stability and rigidity of the hydrogels. As depicted in Figure 1A, Figure 2A, Figure 3A and Figure 4A, the hydrogels are self-supporting, and they remain at the bottom of the inverted vial. Moreover, the fibrillar structure of the hydrogels was confirmed with FESEM observations. The hydrogels comprise a thick network of fibrils with an approximate width of around 10 nm for individual fibrils, which increases when additional fibrils are bundled together (Figure 1B, Figure 2B, Figure 3B and Figure 4B). To further evaluate the extrusion properties of the hydrogels, a portion of an as-yet non-self-assembled gel mixture was transferred into a 1 mL syringe. The peptide–porphyrin mixture was left to self-assemble within the syringe into a clear hydrogel and the syringe tip was removed. The hydrogel was extruded onto a microscopy slide and the stability of the hydrogel was observed over time. The cylindrical shape of the hydrogel was kept intact, and no gel deformation or porphyrin leaks were observed, indicating that the gel integrity was maintained (Figure 1C, Figure 2C, Figure 3C and Figure 4C). 

#### 3.1.2. Evaluation of Hydrogel Mechanical Properties

**Figure 5:**
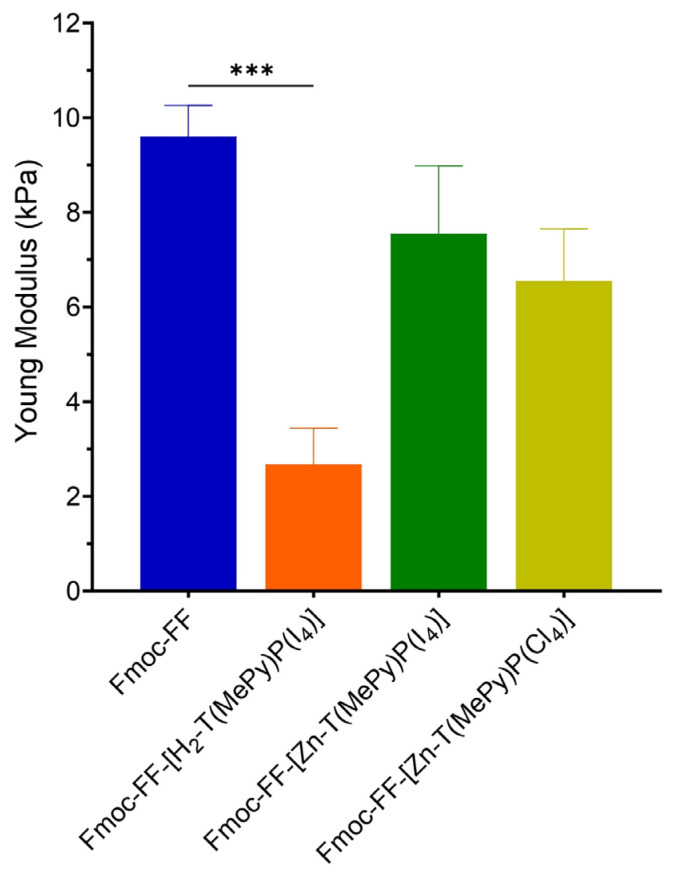
Determination of Young’s modulus of the different hydrogels. Statistical analysis was performed compared to the control hydrogel Fmoc-FF using one-way ANOVA (*** *p* < 0.001).

Young’s modulus provides insights into the material’s ability to resist deformation under stress. Young’s modulus measurements were obtained through uniaxial compression tests (Figure 5) [45,46]. The hydrogels made from Fmoc-FF (control) exhibited a Young’s modulus value of 9.6 ± 0.7 kPa, indicating mechanically stable structures in accordance with the values reported in the literature [42]. The Fmoc-FF-[Zn-T(MePy)P(I_4_)] hydrogels displayed a similar Young’s modulus value of 9.5 ± 1.4 kPa. The Fmoc-FF-[H_2_-T(MePy)P(I_4_)] hydrogel demonstrated a significantly lower Young’s modulus value of 2.3 ± 0.8 kPa compared to the control. These findings suggest that the complexation of Zn inside the porphyrin ring had a substantial impact on the stiffness of the hydrogel, resulting in a stronger material compared to the non-metalated derivative, with a Young’s modulus mirroring the one of the Fmoc-FF. The Fmoc-FF-[Zn-T(MePy)P(Cl_4_)] hydrogel showed a Young’s modulus value of 6.6 ± 1.1 kPa, which was not significantly different from the control.

#### 3.1.3. The Peptide Hydrogels Encapsulating Porphyrins Are Not Toxic to Mammalian Cells

The cytotoxicity of the Fmoc-FF hydrogels loaded with porphyrins was tested for their biocompatibility with mammalian cell lines in order to ascertain their subsequent suitability as therapeutic agents. Initially, the cytotoxicity of the peptide Fmoc-FF hydrogel without any loaded porphyrin was tested with the MTT assay in the NIH3T3 mouse skin fibroblast cell line. No cytotoxicity of the peptide Fmoc-FF has been reported so far; in the present study, it was important to evaluate whether the combination of the solvents used for dissolving the peptide powder and for the self-assembly of the fibrillar hydrogel provide a safe and non-cytotoxic combination.

**Figure 6:**
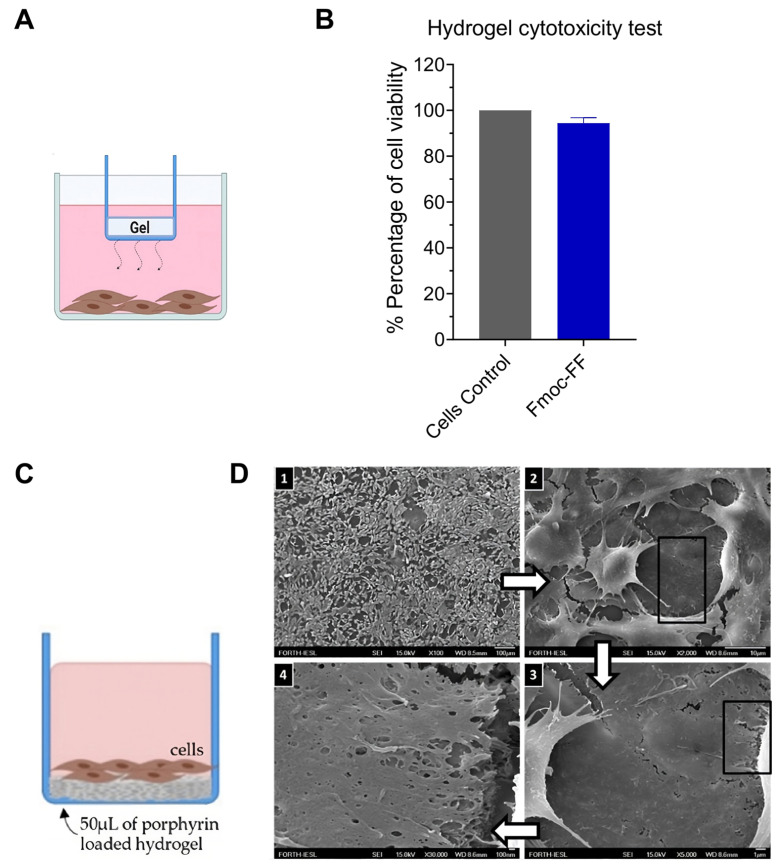
(**A**) Experimental setup for the cytotoxic evaluation of the hydrogels through an indirect contact with the cells. A total of 100 μL of Fmoc-FF hydrogel was placed into a TC insert. The hydrogel was in contact with the cell media through the 0.4 μm porous membrane of the insert. NIH3T3 fibroblasts were previously incubated for 24 h at 37 °C at the bottom of the well; (**B**) MTT cytotoxicity assay results; (**C**) experimental setup of the direct method of testing the cytocompatibility of the H_2_-T(MePy)P(I_4_)-loaded hydrogels. A portion of H_2_-T(MePy)P(I_4_)-loaded Fmoc-FF hydrogel was added at the bottom of a 24-well plate and cells were added on top for a 48 h incubation; (**D**) FESEM observations of the cell proliferation on top of the porphyrin-loaded hydrogels. The same area is scanned with increased magnification. The black rectangles indicate the area that was further magnified. Scale bars for the pictures B1: 100 μm, B2: 10 μm, B3: 1 μm, B4: 100 nm.

The Fmoc-FF hydrogels were formed inside a TC insert and were placed on top of a 24-well plate, where NIH3T3 cells were cultivated for 2 h. The gel was in indirect contact with the cells through the media buffer as depicted in Figure 6A. The hydrogels were incubated in this setup for 48 h at 37 °C, showing no cytotoxic action to the fibroblast cell line (Figure 6B).

In a more direct approach, Fmoc-FF hydrogels encapsulating the H_2_-T(MePy)P(I_4_) porphyrin were added at the bottom of a 24-well plate. NIH3T3 fibroblasts were added on top, and the samples were incubated at 37 °C for 48 h to evaluate the cell proliferation on the hydrogels (Figure 6C). After incubation, the samples were fixed with paraformaldehyde, dehydrated, and placed for FESEM observation. It is concluded from the pictures obtained that the hydrogels are biocompatible and allow cell proliferation (Figure 6D1). Starting from the pictures with lower to higher magnification, it is obvious that the hydrogels provide a biocompatible environment and cells are multiplying while being able to attach and spread on top of the hydrogel (Figure 6D2). At higher magnifications of 1 μm–100 nm, the thick and fibrillar network of the hydrogel that acts as a scaffold for the fibroblast cells can be observed (Figure 6D3, D4). The same experiment was performed for all the combinations of the metalloporphyrin-loaded hydrogels, resulting in a similar outcome (results not shown).

#### 3.1.4. Live/Dead Assay

**Figure 7:**
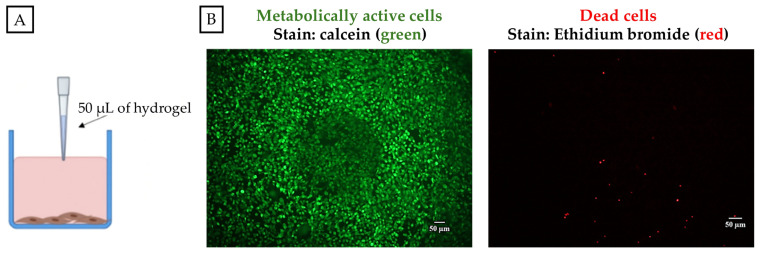
(**A**): Experimental setup for the Live/Dead cytotoxicity assay. The peptide hydrogel was directly added to pre-incubated NIH3T3 cells; (**B**) cell-hydrogel samples were stained and observed under a fluorescence microscope. Left: Exc 494/Em517 nm (green) for the calcein-green filter which detects live cells and right: the 528/617 nm filter for ethidium homodimer-1 for the dead cells.

The Live/Dead cytotoxicity test was also employed for testing the cytocompatibility of the peptide hydrogels. A portion of the self-assembled peptide hydrogel was added to a 24-well plate which already contained attached NIH3T3 cells (Figure 7A). After a 24 h incubation, the cells were stained with calcein-green for detecting the metabolically active cells and with ethidium homodimer-1 for detecting the dead cells. The results confirm the lack of cytotoxicity of the peptide hydrogels since most of the cells produced an intense green color when the filter of calcein was used (live cells) in comparison to a few faint red color spots (dead cells) produced when the ethidium homodimer-I filter was used (Figure 7B).

#### 3.1.5. *In Vitro* Degradation of Hydrogels

**Figure 8:**
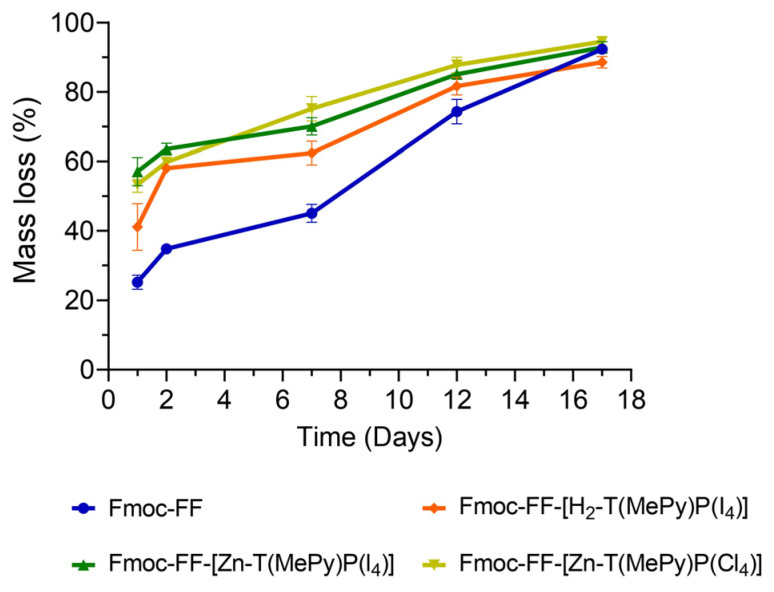
Evaluation of the *in vitro* degradation of Fmoc-FF hydrogel and Fmoc-FF hydrogels with encapsulated H_2_-T(MePy)P(I_4_), Zn-T(MePy)P(I_4_), and Zn-T(MePy)P(Cl_4_) porphyrins at 37 °C. Each bar represents the mean ± SD (*n* = 3–5).

In order to assess the degradation rate of the peptide hydrogels in vitro, their mass loss was investigated after immersion in PBS at 1, 2, 7, 12, and 17 days (Figure 8). The Fmoc-FF hydrogel showed the lowest degradation percentage with a mass loss of 25 ± 2%, followed by 40 ± 7% of the Fmoc-FF-[H_2_-T(MePy)P(I_4_)] hydrogel within 24 h. At day 2, their mass loss increased by an additional 15 ± 1% and 20 ± 1%, respectively. However, the Fmoc-FF-[Zn-T(MePy)P(I_4_)] and Fmoc-FF-[Zn-T(MePy)P(Cl_4_)] hydrogels exhibited a higher degradation percentage on the first day, reaching approximately 55 ± 2% and 60 ± 4%, respectively, and finally reached 93 ± 2% on day 17. The Fmoc-FF-[H_2_-T(MePy)P(I_4_)] hydrogel reached a degradation percentage of 90 ± 2%, while the Fmoc-FF hydrogel reached a maximum mass loss of 90 ± 3% on day 17.

#### 3.1.6. Evaluation of Porphyrin Release Properties from the Hydrogels

**Figure 9:**
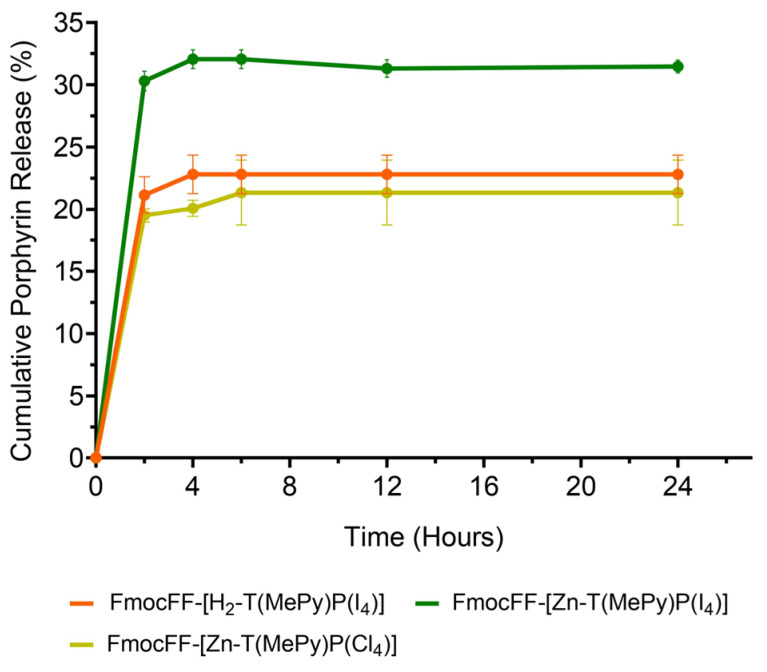
Cumulative release (%) of the H_2_-T(MePy)P(I_4_), Zn-T(MePy)P(I_4_), and Zn-T(MePy)P(Cl_4_) porphyrins from the Fmoc-FF hydrogel at 37 °C.

The release rate of the H_2_-T(MePy)P(I_4_), Zn-T(MePy)P(I_4_), and Zn-T(MePy)P(Cl_4_) porphyrins, which were encapsulated within the Fmoc-FF hydrogels, was studied at the physiological temperature of 37 °C (Figure 9). In all examined cases, an initial burst release of porphyrin derivatives was observed during the first two hours, followed by a gradual release. The initial burst could be attributed to the rapid hydrogel swelling which favors the diffusion of porphyrin. From the Fmoc-FF hydrogel scaffold, we observed that about 20% of the H_2_-T(MePy)P(I_4_) and Zn-T(MePy)P(Cl_4_) porphyrins was being released within 24 h, whereas an increased release of 30% of the Zn-T(MePy)P(I_4_) was observed.

#### 3.1.7. Antibacterial Photodynamic Activity *In Vitro*

**Figure 10:**
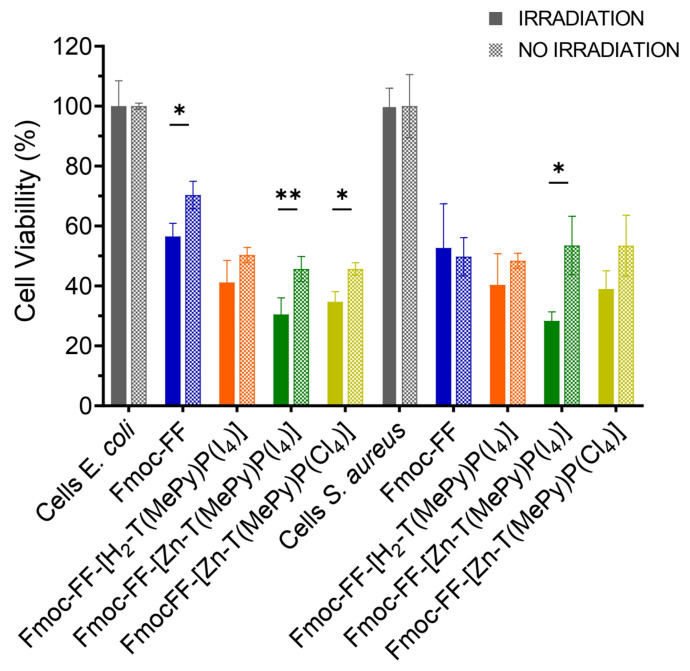
Plot of the cell viability of *E. coli* and *S. aureus* incubated with the Fmoc-FF hydrogel and the Fmoc-FF with encapsulated porphyrins H_2_-T(MePy)P(I_4_), Zn-T(MePy)P(I_4_), and Zn-T(MePy)P(Cl_4_), with and without light irradiation. * *p* ≤ 0.05, ** *p* ≤ 0.01. Two-way ANOVA. The experiments were carried out 3 times in triplicate (*n* = 9).

Demonstrating the negligible cytotoxic impact of all the formed Fmoc-FF hydrogels, we proceeded to examine *in vitro* the effects of the H_2_-T(MePy)P(I_4_), Zn-T(MePy)P(I_4_), and Zn-T(MePy)P(Cl_4_) porphyrins against *E. coli* and *S. aureus,* with and without light activation. As the experimental set-up, we opted to immerse the gel in liquid bacterial cultures that reached mid-log phase at 37 °C and apply irradiation after 5 h, where the porphyrin release reached a plateau. Withdrawing samples after 6 h, plating, and counting the colonies allowed quantitative results to be obtained. The results are shown in Figure 10. We observed that the peptide gel alone (i.e., without encapsulated porphyrins—hence, serving as control group), once exposed to *E. coli* cultures, caused significant inhibition to the bacterial survival with and without light irradiation. In particular, the sole Fmoc-FF peptide reduced the surviving population by 30%, without light irradiation. Exposure to visible LED light (10 mW·cm^−2^, 1 h, light dose of 36 J·cm^−2^) yielded a further reduction of around 13%, strengthening the observation that the Fmoc-protecting dipeptide has a significant effect on bacterial survival. The encapsulation of porphyrins H_2_-T(MePy)P(I_4_), Zn-T(MePy)P(I_4_), and Zn-T(MePy)P(Cl_4_) into the Fmoc-FF scaffold reduced the survival rate to 50%, in the dark. In comparison, the H_2_-T(MePy)P(I_4_) porphyrin showed a further decrease of 9%, under visible light. Similarly, the metalloporphyrin Zn-T(MePy)P(Cl_4_) further reduced the survival rate of *E. coli* by an additional 12%. The similarly mild effect of the porphyrins H_2_-T(MePy)P(I_4_) and Zn-T(MePy)P(Cl_4_) in the Fmoc-FF scaffold could be explained by the fact that they followed the same release kinetics, according to which a significant fraction remains trapped in the hydrogel. Of note, the additional ~10% porphyrin release by Zn-T(MePy)P(I_4_) (with respect to the other two porphyrins) is reflected in an additional 15% reduction in survivability rate.

In experiments like those conducted with *E. coli,* the peptide Fmoc-FF by itself led to a significant 50% reduction in *S. aureus* survival, both with and without light exposure. When *S. aureus* cells were treated with the Fmoc-FF scaffold encapsulating the H_2_-T(MePy)P(I_4_) porphyrin, a comparable 45–50% decrease in survival was noted, regardless of light conditions. These findings suggest that the amount of the H_2_-T(MePy)P(I_4_) porphyrin released into the medium does not significantly amplify its activity upon light exposure and exhibits similar efficacy against both microbial strains. Interestingly, Zn-T(MePy)P(Cl_4_) reduced the survival rate to 50% in the dark, mirroring the effect of the Fmoc-FF peptide alone. When exposed to light, there was an enhanced reduction compared to the porphyrin H_2_-T(MePy)P(I_4_). Given that the release amount of the porphyrin remains consistent, the superior efficacy of the zinc-centered porphyrin becomes evident. Notably, the additional ~10% release of Zn-T(MePy)P(I_4_) relative to the other two porphyrins corresponds to the 35% survival rate observed for *S. aureus*.

## 4. Discussion

Antimicrobial resistance poses a significant global health risk, due to the inappropriate usage of antibiotics [1]. Porphyrins and metalloporphyrins are a class of compounds that have recently been intensively studied as alternative antimicrobial compounds due to their bactericidal properties upon light irradiation [47]. When vehicles for the controlled release of such compounds are needed, peptide-based hydrogels can be chosen, since they have been reported as promising drug delivery agents [48]. The research reported here proposes the encapsulation of porphyrins into peptide hydrogels as delivery vehicles to impart the gels with antimicrobial activity upon light irradiation. The self-assembling Fmoc-FF dipeptide is a well-studied gelator and was selected as a scaffold due to its previously reported biocompatibility. Additionally, the non-metalated porphyrin H_2_-T(MePy)P(I_4_) and its corresponding zinc-metalated analogs were employed as photosensitizers. This research was prompted by the recently demonstrated wound-healing properties of H_2_-T(MePy)P(I_4_) encapsulated into the Fmoc-FF dipeptide, and antimicrobial properties are highly desirable for a wound-healing material [39]. The Fmoc-FF peptide upon encapsulation of the porphyrin derivatives instantaneously self-assembled into hydrogels. The encapsulation of chromophores might influence the self-assembly process of the peptides, potentially affecting the final hydrogel properties. However, as FESEM microscopy confirmed, the fibrillar formation of the hydrogels remained unaffected, suggesting that the porphyrin derivatives did not disturb the inherent structure of the Fmoc-FF hydrogel. Although the structure of the hydrogels remains stable, the mechanical properties change due to porphyrin encapsulation. In the field of medical device design, the stiffness of hydrogels plays a crucial role [45]; native soft tissues and organs generally exhibit Young’s modulus values ranging from 0.1 to 1 MPa [49]. A significant decrease in Young’s modulus was observed with the Fmoc-FF-[H_2_-T(MePy)P(I_4_)] hydrogel compared to the value of 9.6 kPa measured for the Fmoc-FF scaffold. On the other hand, the incorporation of the zinc-metalated porphyrin in the Fmoc-FF-[Zn-T(MePy)P(Cl_4_)] hydrogel did not induce a significant decrease, whereas the Fmoc-FF-[Zn-T(MePy)P(I_4_)] modulus mirrored the one measured for the Fmoc-FF scaffold. These results imply that the introduction of different additives, such as H_2_-T(MePy)P(I_4_) or Zn-T(MePy)P(Cl_4_), into the hydrogel formulation can influence its mechanical properties. The decrease in Young’s modulus observed with the Fmoc-FF-[H_2_-T(MePy)P(I_4_)] hydrogel suggests potential applications requiring a softer material, while the minimal effect on stiffness observed with the Fmoc-FF-[Zn-T(MePy)P(I_4_)] hydrogel may indicate its suitability for applications where stiffer hydrogels are required. These findings contribute to the understanding of hydrogel behavior and aid in the design of tailored materials for specific applications. In hydrogels, a variety of factors such as mesh size, concentration of the encapsulated photosensitizer, dye/drug size, and molecular weight are important for determining the release rate. Moreover, dyes with larger dimensions and larger molecular weights tend to have a limited release rate. Given these insights, it is evident that these parameters can be varied to tailor the release rate and design these hydrogels depending on the drug delivery application required [50]. In the present work, we recorded a relatively weak cumulative release rate of the porphyrins, ranging from 20% to 30%, corresponding to the molar concentrations of 23.4 μM for Fmoc-FF-[Zn-T(MePy)P(Cl_4_)], 25 μM for H_2_-T(MePy)P(I_4_), and 37.5 μM for Fmoc-FF-[Zn-T(MePy)P(I_4_)]. This result can be attributed to the strong electrostatic interactions between the positively charged cationic porphyrins and the negatively charged hydrogel scaffold, probably due to the free carboxy-termini of the Fmoc-FF. Importantly, the prepared hydrogels did not show any cytotoxic effect on the NIH3T3 fibroblasts without light irradiation. This observation aligns with the findings reported in the literature, namely that in the absence of light, cationic porphyrins exhibit negligible cytotoxicity on various human cell lines [51]. Both cytocompatibility and degradability are critical characteristics of hydrogels that determine the efficacy of topical applications, for example, in wound healing [52]. A degradable hydrogel can serve as a temporary template for providing a moist environment that gradually degrades and releases the encapsulated drug to accelerate wound healing. Degradation analysis showed a high degradation rate for all prepared hydrogels over a period of 17 days. The encapsulation of porphyrins was found to increase the degradation percentage of the hydrogels during the first two days. The Fmoc-FF hydrogels with encapsulated porphyrins lost 58% to 66% of their initial mass within 2 days, while the Fmoc-FF hydrogels lost 35±1%. Finally, the Fmoc-FF-[Zn-T(MePy)P(I_4_)] and Fmoc-FF-[Zn-T(MePy)P(Cl_4_)] hydrogels reached a degradation percentage of 93 ± 2% on day 17 compared to the Fmoc-FF-[H_2_-T(MePy)P(I_4_)] hydrogel that reached a degradation percentage of 90 ± 2% and the Fmoc-FF hydrogel that reached a maximum mass loss of 90 ± 3%.

However, significant antimicrobial activity against tested bacterial strains was reported for these porphyrins. The mechanism underlying this antimicrobial efficacy is well-studied and depends on the generation of reactive oxygen species (ROS). This mechanism is influenced by factors including the number of positive charges on the porphyrin, their spatial arrangement at the peripheral positions, and the incorporation of highly hydrophobic aromatic side groups [15].

Prior studies have proved that the Fmoc-FF peptide can exhibit anti-infective potency against multiple bacterial strains due to its aromaticity and surfactant properties [38]. Our data corroborated the Fmoc-FF peptide’s considerable antimicrobial efficiency, displaying superior efficacy against *S. aureus* compared to *E. coli.* This aligns with the results reported in [38], where a significant reduction in *S. aureus* viable biofilm was observed after exposure for 24 h to designer Fmoc peptide hydrogels. As antibiotic-resistant *S. aureus* strains are frequent in nosocomial environments and in biomedical devices (catheters, implants, etc.), the use of Fmoc peptide hydrogels as the basic scaffold for coatings, wound dressings, etc., and eventually combined with other antimicrobial agents to achieve a synergistic effect is very promising. However, despite the plethora of scientific publications concerning Fmoc-FF peptides, no clinical translation has been achieved so far. As correctly pointed out in [38], long-term toxicity studies of the Fmoc gelator motif are needed to achieve this goal. When the H_2_-T(MePy)P(I_4_) porphyrin was encapsulated within the Fmoc-FF scaffold, a comparable 45-50% decrease in survival for both *E. coli* and *S. aureus* strains was recorded, regardless of light conditions. These findings suggest that under the conditions of the experiment, the antibacterial activity of H_2_-T(MePy)P(I_4_) porphyrin is not significantly amplified upon light exposure, and similar efficacy was recorded against both microbial strains. Even under dark conditions, no significant changes in efficiency were observed between *E. coli* and *S. aureus* though some fluctuations with light activation were observed. A number of studies in the last decade have indicated that metallic constituents can modulate the stability and photophysical properties of the porphyrin macrocycle; specifically, Zn (II)-metalloporphyrin complexes exhibit improved efficiency compared to their free base counterparts [8]. Both Zn-T(MePy)P(Ι_4_) and Zn-T(MePy)P(Cl_4_) exhibit higher antibacterial efficiency compared to H_2_-T(MePy)P(I_4_). Notably, these compounds show the highest levels of aPDI inactivation against *E. coli*, with the Zn-T(MePy)P(Ι_4_) exhibiting statistically significant difference between dark and light-irradiated conditions for both *E. coli* and *S. aureus* strains. The Zn-T(MePy)P(Ι_4_) might be the porphyrin of choice for future studies and applications.

## 5. Conclusions

The results presented here confirm that porphyrin-encapsulating Fmoc-FF hydrogels maintain effective self-assembly and mechanical adaptability based on the chosen photosensitizer. The irradiated porphyrin hydrogels exhibited notable efficacy against bacteria, highlighting their antimicrobial potential. Metalloporphyrins, particularly Zn (II)- porphyrin complexes, displayed enhanced bactericidal efficiency compared to their free-base counterpart. Therefore, the combination of a cytocompatible and anti-infective hydrogel scaffold with the antimicrobial efficiency of metalloporphyrins mentioned above creates a potent formulation for topical applications. As described above, we have recently encapsulated the porphyrin H_2_-T(MePy)P(I_4_) into Fmoc-FF hydrogels; their delivery through the self-assembling hydrogel accelerated the healing of experimental skin defects *in vivo* [39]. Therefore, these hydrogels present a promising alternative for both wound care and combating bacterial infections in the face of growing AMR concerns. Moreover, their eventual effect on other pathogens besides bacteria, such as yeasts or viruses, merits to be investigated in the future.

Finally, the results presented here open the way for alternative formulations of Fmoc-FF encapsulating porphyrins for topical use. For example, porphyrin-Fmoc-FF conjugates can be electrospun [53]; therefore, one can envision porphyrin-releasing electrospun mats for antimicrobial applications, for example, wound dressings or for biofilm combatting.

## Data Availability

Data can be made available upon request.

## Abbreviations

AMR	Antimicrobial resistance
Fmoc	Fluorenyl-methoxycarbonyl
H_2_-T(MePy)P(l_4_)	5,10,15,20-tetra-(N-methyl-4-pyridyl) porphyrin tetraiodide
Zn-T(MePy)P(I_4_)	Zinc 5,10,15,20-tetra-(N-methyl-4-pyridyl) porphyrin tetraiodide
Zn-T(MePy)P(Cl_4_)	Zinc 5,10,15,20-tetra-(N-methyl-4-pyridyl) porphyrin tetrachloride
aPDI	Antimicrobial photodynamic inactivation
ROS	Reactive oxygen species
